# HER2 expression in cervical cancer as a potential therapeutic target

**DOI:** 10.1186/1471-2407-4-59

**Published:** 2004-09-01

**Authors:** Alma Chavez-Blanco, Victor Perez-Sanchez, Aurora Gonzalez-Fierro, Teresa Vela-Chavez, Myrna Candelaria, Lucely Cetina, Silvia Vidal, Alfonso Dueñas-Gonzalez

**Affiliations:** 1 Unidad de Investigación Biomédica en Cáncer. Instituto Nacional de Cancerología-Instituto de Investigaciones Biomédicas, UNAM, Av. San Fernando No. 22, Tlalpan 14080, Mexico City; 2 Department of Pathology, Instituto Nacional de Cancerología; Av. San Fernando No. 22, Tlalpan 14080, Mexico City; 3 Division of Clinical Research, Instituto Nacional de Cancerología. Av. San Fernando No. 22, Tlalpan 14080, Mexico City

## Abstract

**Background:**

Trastuzumab, a humanized monoclonal antibody against the HER2 receptor is currently being used in breast and other tumor types. Early studies have shown that a variable proportion of cervical carcinoma tumors overexpress the HER2 receptor as evaluated by diverse techniques and antibodies. Currently it is known that a tumor response to trastuzumab strongly correlates with the level of HER2 expression evaluated by the Hercep Test, thus, it seems desirable to evaluate the status of expression of this receptor using the FDA-approved Hercep Test and grading system to gain insight in the feasibility of using trastuzumab in cervical cancer patients.

**Methods:**

We analyzed a series of cervical cancer cell lines, the primary tumors of 35 cases of cervical cancer patients and four recurrent cases, with the Hercep Test in order to establish whether this tumor type overexpress HER2 at level of 2+/3+ as trastuzumab is currently approved for breast cancer having such level of expression.

**Results:**

The results indicate that only 1 out of 35 primary tumors cases overexpress the receptor at this level, however, two out of four recurrent tumors that tested negative at diagnosis shifted to Hercep Test 2+ and 3+ respectively.

**Conclusions:**

The low frequency of expression in primary cases suggests that trastuzumab could have a limited value for the primary management of cervical cancer patients, however, the finding of "conversion" to Hercep Test 2+ and 3+ of recurrent tumors indicates the need to further evaluate the expression of HER2 in the metastatic and recurrent cases.

## Background

Cervical carcinoma is a leading cause of death in women of reproductive age worldwide, particularly in developing countries. While curable in early stages, the treatment results of locally advanced disease are unsatisfactory. The current standard of treatment -cisplatin-based chemoradiation- fails to cure at least 15% to 45% of bulky IB to IIIB patients, and in addition, multimodality treatment incorporating chemotherapy, surgery and radiation at its best is unlikely to substantially increase the cure rate. Because of this, the logical step to follow is the testing of molecular targeted therapies trying to improve the prognosis of cervical cancer patients [1].

Human papillomavirus infection is recognized as the stronger etiological factor for the development of this tumor; however, overexpression of the epidermal growth factor receptor family members is also common and seems to play an important oncogenic role [2]. HER2 (also known as c-erbB-2) is a transmembrane receptor protein with tyrosine kinase activity that belongs to this family and it is overexpressed in a number of solid tumors. Its overexpression and prognostic significance in breast cancer led to the development and approval of the use of trastuzumab (Trastuzumab, Genentech, South San Francisco, CA), a recombinant monoclonal antibody to HER2, for the treatment of patients with metastatic breast carcinomas overexpressing HER2 [3].

Until more recently, poor standardization in HER2 status evaluation precluded reliable comparison of overexpression rates in different tumors. A source of variability in results not only comes from methodological variations in tissue processing (time to fixation, duration of fixation, denaturation, heating, antigen retrieval, the staining procedure) and grading scores but also from the antibody used. This issue was addressed by Press et al., who showed extremely variable results in 187 breast cancer specimens evaluated with 7 polyclonal and 21 monoclonal antibodies [4]. However, standardized methodologies have been introduced recently for these analyses, and have identified frequencies of 51%, 44%, 26% and 25% in Wilm's tumor, bladder, pancreatic and breast carcinoma, respectively. Other tumors tested had frequencies below 20% [5].

Before the introduction of the Hercep Test, it was known that a variable subset of cervical carcinomas ranging from 8% to 77% express HER2 as evaluated by diverse methods [6-14] and that in some studies its overexpression has shown to confer a worse prognosis [7-9,13]. Because these results on HER2 expression in cervical cancer were obtained before the standardization required in breast cancer, we wanted to investigate the expression status of HER2 using the Hercep Test in a series of cervical carcinoma cell lines, primary tumors of locally advanced cervical cancer cases and in four recurrent tumors of these patients.

## Methods

### Tumor specimens

Thirty-five paraffin-embedded tumor tissues from patients FIGO staged as IB2 to IIIB, treated with standard radiation concurrent with weekly cisplatin. Diagnosis was made on the basis of routine hematoxilin-eosin examination under light microscopy according to the World Health Organization criteria. Tumor specimens at diagnosis were taken before any treatment was instituted whereas the tumors samples from the four recurrent cases were also taken before patients received any second line therapy.

### Cell lines and reagents

DMEM culture media and Fetal Calf Serum were purchased from Gibco BRL Life Technologies (Grand Island, New York). HeLa, CasKi, SiHa and C33A carcinoma cell lines were obtained from the ATCC. The cell line ViBo established from a Mexican patient with cervical cancer was kindly provided by Dr. Monroy (FES Zaragoza, UNAM, Mexico City). Cells were grown in DMEM supplemented with 10% FCS at 37°C and 5% CO_2_. Cell lines were grown on two-chamber polysterene vessel Falcon^® ^(Becton Dickinson, NJ.) and subsequently formalin-fixed for 24 hrs at room temperature, then rehydrated in graded ethanol. Afterwards immunochemistry was performed as below described.

### Hercep test

Hercep Test was performed following the manufacturer's guidelines of HER2 protein expression as follows. Sections were deparaffinized in xylene and rehydrated through graded ethanols to distilled water. The sections were immersed in Dako Epitope Retrieval Solution (10 mM citrate buffer, pH6) that had been preheated to 95°C in a water bath and then heat-treated at 95°C for 40 min. After a 20-minute cooldown period at room temperature, the sections were washed with Dako Wash Buffer, a procedure that followed every subsequent incubation. Endogenous peroxidase was blocked with Dako Blocking Buffer (0.3% hydrogen peroxide containing 15 mM sodium azide) for 5 min at room temperature. The sections were incubated with the primary polyclonal antibody, an affinity-purified rabbit antihuman HER2 antibody supplied in the kit, for 30 min at room temperature. Bound primary antibody was labeled by incubating the slides with the Dako Visualization reagent (horseradish peroxidase-labeled dextran polymer conjugated to affinity-purified goat antirabbit immunoglobulins in Tris-HCl) for 30 min. Color development was achieved with 3,3'-diaminobenzidine (DAB) for 10 min. The sections were counterstained with hematoxylin and eosin. To confirm validation of the staining run, control cell slides, which were provided in the kit and consisted of three pelleted, formalin-fixed, paraffin-embedded human breast cell lines with known HER2 positivity (MDA-231: 0; MDA-175: 1+; SK-BR-3: 3+), were also stained simultaneously. In the negative controls, the primary antibody was replaced by normal rabbit serum (Dako Negative Control Reagent) for the HER2 primary antibody. The antibody used in Hercep Test did not exhibit cross-reactivity to HER3 and HER4 in western blot analysis.

Following the FDA scoring guidelines for breast carcinomas, only membrane staining intensity and pattern were evaluated using the 0–3+ scale as illustrated in the Hercep Test kit scoring guidelines (0 for no staining at all or membrane staining in less than 10% of the tumor cells; 1+ for only partial, weak staining of the cell membrane of more than 10% of the tumor cells; 2+ for moderate staining of the complete cell membrane in more than 10% of the tumor cells; 3+ for intense staining of the complete membrane in more than 10% of the tumor cells). The analysis was performed by a pathologist (VP-S) familiarized in the use of Hercep Test for breast cancer patients. In accordance with the Hercep Test kit guide, HER2 overexpression was assessed as negative for scores of 0 or 1+ and positive for scores of 2+ and 3+.

### FISH in the four recurrent cases

Amplification of *Her-2/neu *was evaluated using the Path-Vysion DNA Probe Kit (Vysis), which uses a dual-color probe for determining the number of copies of both *Her-2/neu *(orange) and the chromosome 17 centromeres (green). The kit was used following the manufacturer's instructions with a few minor modifications. Slides containing 5μ thick paraffin-embedded tissue sections of studied cervical tumor cases and a known Her-2/neu amplified breast tumor were placed on a slide warmer overnight at 58°C, followed by deparaffinization in Xilol, dehydration in 100 ethanol, and drying on a slide warmer at 45 to 50°C. Slides were then pretreated with 0.2 N hydrochloric acid for 20 minutes, followed by washes in purified water and immersion in Vysis wash buffer. They were subsequently immersed in Vysis protease solution at 37°C for 10 minutes, washed in Vysis wash buffer, and dried on the slide warmer. The slides were then immersed in 10% buffered formalin at room temperature for 10 minutes, immersed in Vysis wash buffer, and dried on the slide warmer. Sections were denatured by placing the slides in formamide for 5 minutes at 72°C followed by dehydration in 70, 85 and then 100% ethanol. Slides were then dried on a slide warmer, and 10 μl of probe was applied. They were then coverslipped, sealed and placed in a prewarmed humid incubation chamber at 37°C for 21 hours. This was followed by immersion in prewarmed postwash solution at 72°C for 2 minutes. The slides were air-dried, and a 4',6-diamidino-2-phenylindole (DAPI) counterstain was applied.

The scoring system used is described in detail in the manufacturer's instruction. A minimum of 60 nuclei were scored by each of 2 observers using a Zeiss Axioskop-2 fluorescent microscope with V.2 filter. The ratio of *Her-2/neu *signals (orange) to chromosome 17 centromere signals (green) was determined with ratios of <2.0 considered nonamplified and those ≥ 2.0 amplified.

## Results

The immunohistochemical expression of HER2 in the primary tumors of 35 patients at diagnosis was evaluated. Accordingly, these patients had no received any previous anticancer therapy; their mean age was 40.8 years; 5 were staged as IB2-IIA, 14 as IIB and 16 as IIIB; 31 and 4 were histologically classified as squamous and adeno/adenosquamous respectively. Overexpression of HER2 was demonstrated in only one out of 35 cases, which had a score of 3+ (not shown). The remaining cases were interpreted as negative [score of 0]. The case with HER2 overexpression at diagnosis was a 56-year old woman diagnosed with a FIGO stage IIB large cell poorly differentiated squamous cell carcinoma, who received treatment with 6 weekly courses of cisplatin concurrent with external beam radiation and brachytherapy. She is currently free of disease at 44 months of follow-up. At a median follow-up time 40 months, seven patients have relapsed.

**Figure 1:**
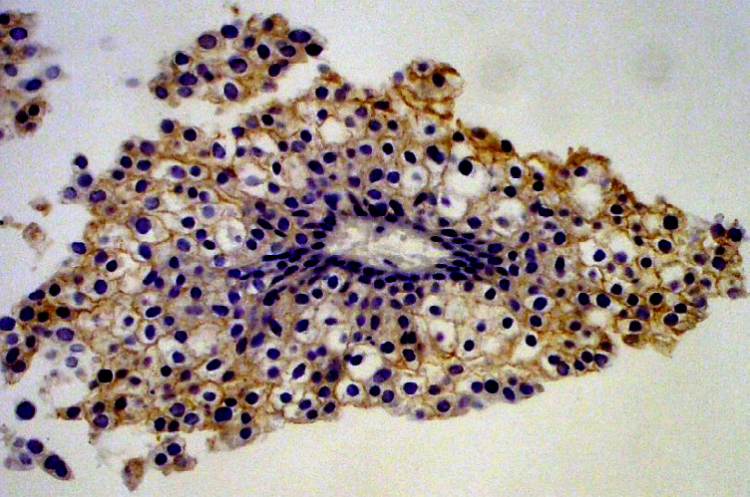
Photomicrography of a recurrent tumor positive at a intensity of 2+. (200X).

**Figure 2:**
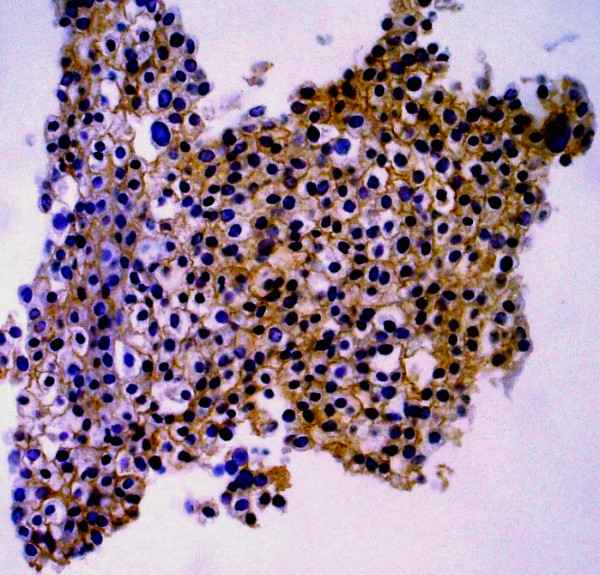
Photomicrography of a recurrent tumor positive at a intensity of 3+. (200X).

HER2 expression at recurrence could only be analyzed in four of these seven relapsed patients in whom whose recurrent disease was histopathologically confirmed. Two of these four tested positive with a staining intensity of 2+ and 3+ respectively, (Figures 1 and 2), both cases were squamous cell carcinomas and tested negative in the pretreatment surgical specimen

The five cervical cancer cell lines were negative.

**Figure 3:**
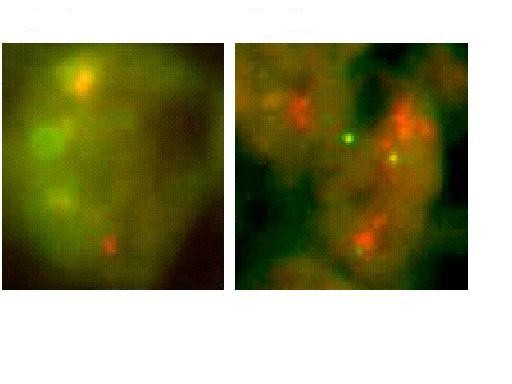
Photomicrography of the FISH analysis of a negative case of a recurrent tumor that was Hercep Test-positive, and a control positive case of breast carcinoma.

None of the four recurrent cases tested by FISH were HER2 amplified (Figure 3).

## Discussion

Molecular targeted therapies are currently being tested in a variety of tumor types with promising results. Because HER2 overexpression occurs and is related to a worse prognosis in cervical cancer, [7-9,13], its blockade with trastuzumab could potentially have therapeutic value. This monoclonal antibody is currently widely used in metastatic breast cancer and is being evaluated in an adjuvant setting as well as in a variety of tumor types [3,15-17], Based on the fact that the efficacy of this antibody is strongly associated to the level of HER2 expression in the primary tumor, the FDA approved the Hercep Test in the aim to grade the expression level so that only those patients whose tumors exhibit a 2+/3+ levels are candidates to trastuzumab therapy, though currently in most centers, tumors expression of 2+ is considered undeterminate therefore these cases are also evaluated by FISH analysis [18].

Previous reports on cervical cancer using non-standardized methods for HER2 expression showed that up to 77% of cases express the receptor and that in general HER2 expression predominates in adenocarcinoma and adenosquamous carcinoma histologies [6-14] In this work, using the Hercep Test with its corresponding guidelines for evaluation, we found contrastating results as none of the cell lines expressed HER2 and only a single tumor of squamous histology (1 out of 35) expresses this oncoprotein at a level of 3+. Such a discrepancy does not seem to be limited to this tumor type. For instances, in ovarian clear cell carcinoma a 43% of overexpression was reported utilizing systems other than Hercep Test [19], however, when evaluated with this standardized test, only 1 out of 17 tumors expressed 3+ [20]. Likewise, the proportion of patients with 2+/3+ expression level of HER2 with the Hercep Test is uniformly low in tumor such as lung [21], colorectal carcinomas [22], gallbladder [23], and melanoma [24]. A variety of factors such as the kind of antibody used, the technique per se, and scoring criteria may explain such phenomenon and its clarification requires further studies. A recent paper by Bellone et al., have reported that a substantially higher proportion of cervical cancer cells lines either established from fresh tumors or commercial ones (including CasKi, SiHa, HeLa and C33A which are negative by immunochemistry) express the receptor when evaluated by flow-cytometry and are growth inhibited when incubated with trastuzumab or trastuzumab plus IL-2 [25]. These data led them to suggest the targeting the HER in cervical cancer could be more effective than the indicated by low immunohistochemical expression [25]. However, it is largely known that in breast [26] and more recently in lung cancer [27], tumor responses are almost confined to those with a 3+ level of expression. For instances, in a recent published study in 111 assessable breast cancer patients, the response rate to single agent trastuzumab for those expressing 3+ versus 2+ was 35% and 0% respectively [26], while in lung cancer, a phase II trial of gemcitabine-cisplatin with or without trastuzumab in HER2-positive patients, yet there was not overall differences in response between both arms, the benefit was limited to those with 3+ of expression with the Hercep Test. Accordingly, five out of six patients with such level of expression receiving trastuzumab plus gemcitabine-cisplatin showed response [27]. These data argue against the potential usefulness of trastuzumab in cervical cancer patients with HER expression that can only be detected by flow cytometry [25].

On the other hand, the HER2 expression in breast cancer is relatively stable, with 95% concordance between the HER2 status of primary and metastatic lesions, being rare a shift from positivity in the primary to negativity in the metastases [5]. Conversely, 6% of breast cancer patients whose primary tumors are HER2 negative, convert to high expression (Hercep Test 3+) in their metastases [28]. Such behavior is in line with experimental observations that receptor activation potentiates tumor cell motility, protease secretion and invasion, and also modulates cell cycle checkpoint function, DNA repair, apoptotic responses and multidrug resistance [29,30]. The findings of "conversion to positive" in cells of recurrent cervical tumors showed by us and other authors [25,31] strongly suggest that expression of HER2 may have a role in tumor resistance and progression as shown in experimental models, and therefore its targeting in recurrent cervical cancer could have therapeutic value.

It is remarkable the finding that none of the four recurrent cases, including the two that converted to IHC positive analyzed by FISH showed HER2 gene amplification. This result is unlikely to be a false negative as the green signal was perfectly observed in most of the cells. Previous studies in cervical cancer have shown that the frequency of gene amplification as determined by FISH is low irrespective of tumor histology. Mark et al., reported only 2 out of 23 cases amplified using the Her-2/neu FISH probe (Vysis, Inc., Downers Grove, IL) both of which were adenocarcinomas [32]. In a more recent study looking at DNA copy number of cervical adenocarcinomas, it was found that despite more than 50% of patients had chromosome 17q copy number gains, only 9% (2 out of 22) of these tumors showed an HER-2/neu protein over-expression at the level of 2+ with the Hercep test. These findings suggest that amplification of HER-2/*neu *is rare in cervical adenocarcinomas and that low level chromosome 17q copy number gains are not associated with HER-2/*neu *overexpression [33]. Such overexpression without gene amplification could result from transcriptional deregulation leading to increased receptor expression [34] and is not a rare phenomenon in breast carcinoma [35].

## Conclusions

In conclusion, our study suggest that the clinical usefulness of anti-HER2 antibodies in the primary treatment of cervical cancer patients may be limited due to the low frequency of HER overexpression, nevertheless, it is desirable to further test a larger number of recurrent cervical cancer patients by IHC and FISH analyses regardless of the histological type, as a start point for clinical trials design using trastuzumab.

## Competing interests

None declared.

## Authors'contributions

A C-B, AG-F, carried out the tissue culture work and immunohistochemical analysis; VP-S and T V-C interpreted the histological data; MC critically analyzed and participated in manuscript; CP, contribute with the clinical data; SV performed the FISH analysis; and AD-G conceived the study and wrote the manuscript. All authors read and approved the final manuscript.

## Pre-publication history

The pre-publication history for this paper can be accessed here:
